# mHealth and global mental health: still waiting for the mH^2^ wedding?

**DOI:** 10.1186/1744-8603-10-17

**Published:** 2014-03-26

**Authors:** Conor Farrington, Angela Aristidou, Kai Ruggeri

**Affiliations:** 1 Cambridge Centre for Health Services Research, Institute of Public Health Forvie Site, University of Cambridge School of Clinical Medicine, Cambridge Biomedical Campus, Box 113, Cambridge CB2 0SR, UK; 2 Judge Business School, University of Cambridge, Cambridge CB2 1BZ, UK; 3 Department of Engineering, University of Cambridge, Cambridge CB2 1BZ, UK

**Keywords:** Global mental health, Mobile phones, mHealth, mH^2^

## Abstract

**Background:**

Two phenomena have become increasingly visible over the past decade: the significant global burden of disease arising from mental illness and the rapid acceleration of mobile phone usage in poorer countries. Mental ill-health accounts for a significant proportion of global disability-adjusted life years (DALYs) and years lived with disability (YLDs), especially in poorer countries where a number of factors combine to exacerbate issues of undertreatment. Yet poorer countries have also witnessed significant investments in, and dramatic expansions of, mobile coverage and usage over the past decade.

**Debate:**

The conjunction of high levels of mental illness and high levels of mobile phone usage in poorer countries highlights the potential for “mH^2^” interventions – i.e. mHealth (mobile technology-based) mental health interventions - to tackle global mental health challenges. However, global mental health movements and initiatives have yet to engage fully with this potential, partly because of scepticism towards technological solutions in general and partly because existing mH^2^ projects in mental health have often taken place in a fragmented, narrowly-focused, and small-scale manner. We argue for a deeper and more sustained engagement with mobile phone technology in the global mental health context, and outline the possible shape of an integrated mH^2^ platform for the diagnosis, treatment, and monitoring of mental health.

**Summary:**

Existing and developing mH^2^ technologies represent an underutilised resource in global mental health. If development, evaluation, and implementation challenges are overcome, an integrated mH^2^ platform would make significant contributions to mental healthcare in multiple settings and contexts.

## Background

Mental health is increasingly (if belatedly) recognised as a pressing global health issue. Mental and behavioural disorders accounted for 7.4% of Disability-Adjusted Life Years (DALYs) worldwide in 2010, an increase of 38% since 1990, while major depressive disorder is now the 11th highest cause of DALYs [1]. Mental and behavioural disorders cause 22.7% of Years Lived with Disability (YLDs) worldwide, the highest burden of any disease category [2]. The challenges of meeting mental health needs are greatest in low to upper-middle income countries (classified according to the World Health Organization groupings, which are based upon the World Bank listing of economies)^a^. Compared to high income countries, these countries collectively face a higher proportion of the global mental health burden with more limited overall financial resources and lower levels of spending on mental healthcare, with strongly negative implications for funding community-based and institutional care, pharmacological treatments, and psychosocial interventions [3]. Table 1 illustrates the misalignment of burden and spending commitments by presenting the most recent WHO statistics on mental health DALYs (2004) alongside government mental health spending (2011) by WHO income group.

**Table 1:** Mental health DALYs and mental health spending by WHO income group

**WHO income group**	Mental health DALYs*	Government mental health spending (median mental health expenditures (USD) per capita)**
Low	19 999	0.2
Lower-Middle	69 653	0.59
Upper-Middle	69 609	3.76
High	40 234	44.84

*2004 figures from World Health Organization [4].

**2011 figures from World Health Organization Mental Health Atlas [5].

These sharp disparities also emerge with regard to mental health workforces. Table 2 illustrates the striking differences in mental health human resources between WHO income regions (especially between countries in the Low to Upper-middle income groups on one hand and the High income group on the other).

**Table 2:** Mental health human resources per 100,000 population by WHO income group in 2011

**WHO income group**	**Psychiatrists**	**Psychologists**	**Psychiatric nurses**	**Social workers**
Low	0.05	0.02	0.42	0.01
Lower-middle	0.54	0.14	2.93	0.13
Upper-middle	2.03	1.47	9.72	0.76
High	8.59	3.79	29.15	2.16

Source: World Health Organization Mental Health Atlas [5].

In addition to underfunding and workforce shortages, a range of other factors combine to place severe limits on mental healthcare delivery in poorer countries worldwide. These factors include: low diagnosis rates; socio-cultural stigmas regarding mental illness, leading to associated human rights abuses; inadequate workforce training and continued professional development structures; a focus on institutional rather than community-based care; a lack of suitable policy frameworks; and a lack of political commitment to prioritising mental healthcare [2,3,6-8]. Undertreatment for mental illness is a significant issue even in wealthier countries, with an estimated 35-50% treatment gap for serious disorders [9]. However, the problem is even more serious in poorer countries, with an estimated treatment gap of 76-85% [9]. In Africa, it has been reported that as few as one in ten of the mentally unwell receive appropriate treatment, while psychiatric help was reported to be the least preferred option for mentally ill people seeking help in India [3,10]. The limited data available suggest furthermore that the mental healthcare treatments that are delivered in poorer countries are inequitably distributed, likely to be of limited effectiveness, and often impose significant economic burdens on patients [2,3,10]. More widely, it is increasingly recognised that mental illness interacts negatively with both poverty and physical illness, with the mentally ill more likely to be poor and physically unwell and vice versa; as such, countries that experience poverty and high levels of physical illness are also likely to experience high levels of mental illness, compounding existing challenges [3,11-13]. Taken together, these factors highlight the significant global inequities and challenges arising in the mental healthcare field.

In response to these challenges, initiatives such as the WHO Mental Health Global Action Programme (mhGAP), Grand Challenges for Mental Health, *The Lancet* series on Global Mental Health (2007 and 2011), and the wider Movement for Global Mental Health have encouraged research, clinical, and policy communities to work towards the provision of effective and affordable mental healthcare in a range of socio-economic contexts [3,6-8,10-15]. These initiatives have raised awareness regarding the effectiveness of existing treatments for mental disorders, the importance of countering stigmas, and the need to build clinical capacity and scale-up service provision through (e.g.) training non-specialist mental health workers. Significant challenges remain: the global burden of mental health is likely to rise rather than fall in the near future, stigmas remain deeply-embedded, and little has yet changed regarding resource allocation for mental healthcare in poorer countries [1,2,16]. The paucity of mental health research conducted in such contexts renders the effectiveness of mental health treatments somewhat uncertain, particularly for some important conditions (e.g. developmental disabilities, alcohol-use disorders) and in some settings (e.g. humanitarian crises), while the socio-cultural stigmas attached to mental illness are echoed at the international level by the ongoing neglect of mental health within the global health movement more broadly [16,17]. Nevertheless, since the baseline for mental healthcare delivery in poorer countries is so low and the burden of mental illness so high, there remains both strong potential and urgent need for significant improvements in mental health around the globe. In this paper we argue that global efforts to bring about such improvements have so far largely overlooked the significant potential of technological solutions in mental healthcare, especially with regard to mobile telephones.

## Debate

### mH^2^: promising but overlooked

Is everything being done that could be done? Arguably, the global mental health movement has yet to engage fully with the potential contributions of emerging technologies in the field of mHealth - i.e. the use of mobile communication devices, especially mobile phones (cellphones and/or smartphones), to support and/or deliver healthcare. The various initiatives mentioned above focus largely on practical, low-cost, and easy-to-use solutions – a focus that is often assumed to exclude technological solutions. For example, only one of the 25 Grand Challenges in Mental Health identified by Delphi panel members refers explicitly to technology (although several of the other challenges could be realised via technological means) [13]. Similarly, the Director-General of the WHO states in her introduction to the mhGAP interventions guide for mental, neurological, and substance use disorders that ‘[t]here is a widely shared but mistaken idea that improvements in mental health require sophisticated and expensive technologies’ whereas the reality (she believes) is that ‘[w]hat is required is increasing the capacity of the primary health care system for delivery of an integrated package of care by training, support and supervision’ [14]. Prominent reviews and journal series call for new modes of care delivery, but, with the exception of occasional passing references to the general need for technological advancement in the mental health field, mostly omit any discussion of the potential role of mHealth in this regard [2,13]. As one prominent blogger puts it, we are still ‘waiting for the wedding’ between global mental health and technology [18] – or, as we put it, the mH^2^ wedding (with mH^2^ signifying the synergy between two kinds of ‘m’ health - mHealth and mental health).

This cautious approach to technology, and a concomitant assumption that all technology is inevitably ‘sophisticated and expensive’, overlooks the possibility that affordable mH^2^ systems could aid the delivery of interventions with known effectiveness (such as cognitive behavioural therapy, or CBT) by capitalising on the dramatic expansion in cellphone and, increasingly, smartphone usage in poorer countries. Around three-quarters of the world now has access to a mobile phone, with 77% of mobile subscriptions located in poorer countries in 2010 (see Table 3 below) with poorer countries leapfrogging Western telecommunications trajectories to follow 'mobile first' patterns of development [19]. Moreover, as the availability of smartphones increases, costs fall, and network bandwidths expand, mobile capabilities will play increasingly significant roles in everyday life [19,20].

**Table 3:** Mental health DALYs, mobile phone subscriptions, and growth in mobile phone subscriptions by United Nations Millennium Development Goals (UNMDG) groups

**UNMDG groups**	Total mental health DALYs 2004*	Total mobile phone subscriptions 2012 (millions)**	Average growth in mobile phone subscriptions 2008–2012 (%) ***
Developed Regions	40 715	1 522.33	10.15
All regions other than Developed Regions	159 730.85	4 735.94	46.29^****^
Northern Africa	4 334	195.94	36.64
Sub-Saharan Africa	20 849	537.83	53.89
Latin America and the Caribbean	20 926	657.7	27.64
Caucasus and Central Asia	2 403	84.43	48.18
Eastern Asia	39 196	1 158.7	34.81
Southern Asia	50 200	1 196.03	75.07
South-eastern Asia	15 998	684.62	49.35
Western Asia	5 603	216.56	41.3
Oceania	221.85	4.13	49.7

*2004 figures from WHO [4].

**2012 figures from World Bank, which defines mobile phone subscriptions as follows: ‘subscriptions to a public mobile telephone service using cellular technology, which provide access to the public switched telephone network. Post-paid and prepaid subscriptions are included [21]’.

***Average of growth of mobile subscriptions in constituent countries in each grouping; not weighted by population. See Additional file 1 for statistics for mental health DALYs and mobile subscriptions (2008–2012) by UNMDG group and individual country (figures from WHO and World Bank [4,21]).

****Average of growth across non-developed region UNMDG groups; not weighted by population size or number of constituent countries in each group.

Regarding mHealth in particular, mobile phone ubiquity helps to overcome entry barriers to healthcare, especially for remote and poorer populations, and offers significant potential for remote diagnosis, monitoring, and treatment, in addition to remote training (mLearning) for nonspecialist healthcare workers. The potential for mHealth solutions is particularly strong in mental health since many treatments for mental illness (e.g. CBT) can be delivered with greater ease to patients in situ via mobile phone SMS, voice and interactive multimedia capacities than is the case for most physical disorders, and since the ability to engage in mental treatment remotely and anonymously may help to avoid socio-cultural stigmas regarding mental illness [22,23]. It is also possible, though yet to be confirmed by research, that the high status typically associated with new technological devices such as smartphones may assist efforts to counter the stigma of mental illness, both by encouraging patients to engage with healthcare services and by helping to transform societal preconceptions about mental health through the treatment of mental illness via ‘prestigious’ technological devices.

In short, the conjunction in poorer regions of high mental illness burdens and low levels of service provision on the one hand and relatively high levels of mobile coverage on the other highlights the potential to address the former via the latter. Table 3 illustrates the potential of mH^2^ interventions by juxtaposing World Bank figures regarding mobile subscriptions with WHO mental health DALY statistics grouped according to the United Nations Millennium Development Goals (UNMDG) categories, which allow for greater differentiation between geographical contexts than WHO income group or regional categorisations^b^.

Assuming that mobile phone subscriptions can be taken as an approximate indication of the potential penetration and impact of mH^2^ interventions, Table 3 can be interpreted as illustrating the significant potential synergies that exist between mHealth and mental health, especially in areas such as Eastern and Southern Asia, which demonstrate both a high mental health burden and high numbers of mobile subscriptions in 2012 (owing partly to the inclusion of China in ‘Eastern Asia’ and India in ‘Southern Asia’). Table 3 also illustrates the striking preponderance of mental health DALYs, absolute numbers of mobile phone subscriptions, and high rates of growth in mobile phone subscriptions in the ‘developing’ world (i.e. all regions other than the ‘developed’ world), emphasising simultaneously the ‘mobile first’ development of telecommunications in poorer countries and the concentration of the mental health burden in those areas of the world with the least capacity to address it via conventional (i.e. non-mH^2^-facilitated) mental healthcare (see Tables 1 and 2). Compared to an average 2008–2012 growth rate of mobile subscriptions of just 10.15% in the developed region, some poorer countries demonstrate extremely high growth rates, e.g. Zimbabwe (86.88%), Ethiopia (90.48%), Myanmar (93.25%), and Bhutan (394.59%, albeit in this case from an extremely low base; see Additional file 1 for details on individual country figures). At the same time, the still-significant mental health burden in the developed world and relatively high numbers of mobile subscriptions, considered alongside the aforementioned treatment gap of up to 50% in wealthier countries, also highlights the potential to deliver mental healthcare via mH^2^ solutions in this region as well as in poorer countries.

### mH^2^: a nascent field

In terms of existing work in the mH^2^ field, a small but burgeoning research literature attests to the effectiveness and acceptability of a range of mH^2^ interventions, ranging from the collection of ecologically valid patient information to the use of automated, downloadable mental health apps, and from large-scale SMS public mental health promotion schemes to small-scale, sophisticated systems utilising smartphone sensors to generate context-aware support for real-time interventions and medication adherence, in addition to the well-tried expedient of delivering information or therapeutic treatment via voice-calls [20,22-31]. Prominent examples of mH^2^ systems include: the Mobile Mood Diary, [24] which allows users to report energy, mood, and sleep levels, and which demonstrated increased patient adherence to mood charting compared to paper-based charting; the Mobilyze! system for unipolar depression [25], in which 38 concurrent phone sensor values were utilised by machine learning algorithms to predict users’ mood, emotions, activities, and cognitive states, with preliminary evidence of efficacy and improvements in self-reported depressive symptoms; and the MONARCA self-assessment system for bipolar depression, [26] incorporating features of the Mobile Mood Diary and Mobilyze! systems alongside an inbuilt trigger and notification system, and exhibiting improved self-assessment adherence and high perceived usefulness. Ongoing work by Ekberg and colleagues focuses on the delivery of CBT via cellphones for anxiety disorders, while Vogel and colleagues have demonstrated the effectiveness of cellphone-delivered CBT for obsessive-compulsive disorder [22,27].

While these studies represent important contributions to the developing mH^2^ field, there are at present no systems that attempt to provide an integrated platform capable of utilising the full range of mobile functionality for the treatment of multiple mental health disorders (as opposed to individual conditions such as unipolar or bipolar depressive disorders, or obsessive-compulsive disorder) in a range of global settings. Arguably, this fragmentation of research effort, with research and development projects focusing on narrow aspects of technology and mental health rather than developing wider systems capable of addressing a range of disorders through a range of mobile capabilities and in a range of contexts, has limited the impact of mH^2^ interventions in the global mental health context [19,31]. Moreover, while an increasing number of studies of systems such as Mobilyze! and MONARCA report positive findings, they typically feature small numbers of participants (e.g. 8 for Mobilyze! and 12 for MONARCA) over relatively small periods of time (8 weeks for Mobilyze! and 14 weeks for MONARCA), and in single-arm pilot studies rather than randomised controlled trials. While such limitations are probably inevitable in nascent areas of research funded by short-term academic grants, there is a need nonetheless not just for more ambitious and integrated mH^2^ systems, but also for more extensive, long-term, and rigorous attempts to evaluate the effectiveness and acceptability of such systems in the mental health context (as with mHealth more generally [32]). Once these features of this emerging field are taken into account, it is perhaps less surprising that the global mental health movement has yet to take full account of the mobile revolution constituted by the emerging systems, capabilities and potentialities discussed above.

### The way forward? an integrated mH^2^ platform

We have suggested in this paper that there is room for a more ambitious, integrated, and rigorous approach to developing mH^2^ systems for global mental healthcare. How might this approach look? Designing, piloting, and rigorously testing an effective, and importantly a cost-effective, mH^2^ system capable of meeting the needs of users in a range of contexts with a range of disorders would require interdisciplinary collaboration across a range of academic communities including psychiatry, psychology, global health, IT, and social science, in addition to stakeholders in other contexts such as policymakers, industry, and telecommunications. At minimum, such a system would need to meet the following criteria: build on existing research in the mH^2^ field, as discussed above; incorporate preventative, diagnostic, monitoring, therapeutic, and educational functions, in line with the best available evidence; be culturally adaptable and interoperable with existing face-to-face healthcare systems (e.g. with regard to healthcare records) in addition to dedicated interactive e-Health (i.e. internet-based) programmes; incorporate public-private partnerships and sustainable business models (including mobile payment systems), alongside adequate security protection [33]; draw upon the full range of mobile capabilities, from SMS to voice-calls to the latest smartphone sensors, in order to serve the widest possible range of users and disorders; train mental healthcare professionals and lay workers through mLearning (and eLearning) programmes, in line with recent calls for increased attention to lay community mental health workers in order to expand the overall pool of mental health knowledge and expertise [34]; and provide a range of accessible and up-to-date mental health resources for both healthcare professionals and patients.

Throughout development, testing, and implementation processes, mH^2^ interventions should recognise that mental health diagnostic and therapeutic approaches developed in high-income countries (e.g. psychometric measures and psychological therapies such as CBT) may have varied and/or limited relevance in the diverse social, cultural, economic, political and geographical contexts of middle- and lower income countries. Pilot studies and evaluation of the system regarding different disorders and user groups should be followed by randomised controlled trials in a range of geographical contexts to build a strong evidence-base regarding levels of effectiveness over time and across cultures, including consideration of potential variations in acceptability and cost-effectiveness in the context of different healthcare systems and societal settings. Considerations of cost-effectiveness are important given that an ambitious project of this scale would require significant funding on both an initial and ongoing basis, in order to establish a viable platform and subsequently to modify and update the platform in line with individual socio-cultural settings and technological progress and change.

To facilitate cultural adaptability, system development should proceed in light of active engagement with a range of stakeholders in each geographical context in order to benefit from stakeholder insights (especially early adopter insights) and enable local adaptability. Relevant stakeholders will typically include policymakers and managers, healthcare professionals, community workers, and individual end users (i.e. patients). By according due weight to local interests, realities, preferences, cultural factors, and societal topics of concern, this approach will aid the development of locally-tailored interventions within the broader integrated platform umbrella. Moreover, since stakeholder engagement in product development typically strengthens user motivation, generates perceptions of ownership, and facilitates technology adoption [35], user involvement will help to maximise the acceptability of mH^2^ interventions while also helping to contain and/or avoid some of the risks associated with extensive investment in unknown markets. For this reason, academic and/or corporate researchers in the mH^2^ arena should identify realistic target populations along multiple axes (including age, gender, ethnicity, socio-economic status, and geographical location) and involve these populations in product development and user testing, in order to ensure that (e.g.) older users are well-motivated and able to interact effectively with the mobile technology that facilitates mH^2^ interventions [36]. Consequently, while the envisaged integrated platform will operate on a global basis, the particular shape of the platform – i.e. the characteristics and emphases of the various user interfaces made available to professionals and patients - is likely to vary substantially between contexts.

It should also be acknowledged that some implementation challenges are likely to range beyond the obvious need to tailor generic mH^2^ interfaces and interventions to local circumstances. Technologies are always embedded in social, cultural, economic, political, environmental, geographical, and interpersonal settings, with the consequence that the successful development, implementation, adoption, use, and evaluation of any particular technology (including mH^2^ technologies) requires careful negotiation of complex social realities [37,38]. These challenges are heightened by contrasts between wealthier and poorer countries, particularly (as noted previously) given the urgent need for more mental health research in poorer countries. While recent research conducted in Western countries suggest positive community attitudes towards mH^2^ interventions [39], it would be unwise to assume that the same would hold true in diverse settings worldwide, especially given the arguably greater prevalence of stigma and lack of knowledge regarding mental health in poorer countries. The rationale for, and specific content of, mH^2^ interventions may conflict with specific cultural and religious understandings of mental illness and associated issues such as appropriate mental health treatments, raising the possibility of conflicts between community attitudes and mH^2^ innovations in mental healthcare. It is also important to recognise that mobile usage is often unevenly spread and that many individuals and some social groups utilise mobile telephones on a systematically lower basis than others [20], raising the possibility of unequal access to locally-tailored mH^2^ interventions. Furthermore, the process of merging integrated mH^2^ platforms with existing local mental healthcare systems is likely to encounter resistance, or at least scepticism, indifference, and low levels of motivation to change, on the part of stakeholders with vested interest in the status quo, including healthcare professionals, policymakers, and politicians (as has already become obvious in terms of widespread reluctance to increase mental health spending and/or transition to community care models [3]).

While this array of challenges is undoubtedly significant and requires serious consideration, there are indications that they are not likely to be insurmountable. For example, existing research suggests that diagnostic and therapeutic mental health methodologies are effective in poorer country contexts despite originating in higher income countries [17,40,41]. With specific regard to mH^2^ interventions, scholars have begun to evaluate levels of acceptability of such interventions in community settings both in non-Western contexts and for marginal groups in wealthier countries (e.g. Hispanic farm workers in the USA) [34,42,43], drawing tentatively positive conclusions that point towards the strong, if as yet largely unrealised, potential of mH^2^ interventions in a range of contexts and for a range of user groups. mLearning and eLearning programmes, moreover, have the potential to widen and deepen the pool of (mental) health expertise on the part of both professionals and patients [44,45]. In terms of unequal access to mobile phones, accelerating mobile usage in poorer countries (Table 3) and the introduction of more affordable, locally-developed smartphones is likely to significantly broaden and deepen access to mH^2^ solutions (and technological competence and expertise more widely) over coming years and decades [46] – a phenomenon that also points towards the possibility of contesting mental health stigma, lack of knowledge, and community resistance both through use of mH^2^ interventions and through mobile technology-facilitated versions of existing psycho-education programmes [17]. Vested interests on the part of policymakers, politicians and healthcare professionals are typically more difficult to overcome, with professionals in particular demonstrating strong adherence to established organisational norms and a consequent unwillingness to change [47,48]. However, it is possible that vested interests and a lack of motivation to accept change can be accommodated and at least partially overcome through education and training and by involving stakeholders in co-design processes (as mentioned above), alongside high-level attempts to generate political will in favour of mH^2^ interventions (as with global mental health more generally [7]).

If the various challenges facing mH^2^ interventions are addressed with sufficient care and vigour, it seems reasonable to suggest that a validated system (or systems) of the kind outlined above, if tailored effectively to local conditions and integrated into wider global efforts to destigmatise, deinstitutionalise and scale up mental healthcare, could revolutionise mental healthcare and deliver significant impacts on outcomes worldwide, particularly (but not exclusively) in poorer countries with high levels of both undertreatment and mobile coverage. The successful implementation of mH^2^ systems would also help to address the current paucity of global mental health research [16,17] by generating rich quantitative and qualitative research data on a range of important topics, including: the specific characteristics of mental disorders in poorer countries; cultural, socio-economic, and geographical variations in the acceptability and efficacy of mH^2^ interventions themselves; the effectiveness of mLearning educational programmes for lay mental health workers; and the impact of mH^2^ interventions on stigmas surrounding mental health. Lastly, it is also worth noting that there is also considerable potential for mH^2^ solutions in ‘extreme’ contexts with recognised psychological challenges, such as manned spaceflight, submarine navigation, and Antarctic exploration [49]. Consequently, while the challenges of bringing about the mH^2^ wedding across multiple contexts are significant, the potential rewards are greater still.

### Summary

A range of policy, clinical, and research initiatives have emerged in response to the challenges associated with global mental health. However, these initiatives have yet to take full account of the potential contributions of emerging technologies in the mH^2^ field, especially in poorer countries with high levels of mental illness and mobile phone usage alongside low mental healthcare spending and low workforce levels. A growing literature attests to the effectiveness of mH^2^ interventions utilising a range of mobile phone capabilities, but the ongoing fragmentation of research effort and the inadequate weight of evidence has limited the impact of these developments. We advocate the development of an integrated and rigorously tested mH^2^ platform in order to provide diagnostic, monitoring, and therapeutic interventions in mental health in addition to providing resources and training for mental health workers worldwide.

## Endnotes

^a^The composition of these groups is available online at: http://www.who.int/healthinfo/global_burden_disease/definition_regions/en/index.html.

^b^UNMDG Country Groupings available at: http://mdgs.un.org/unsd/mdg/Host.aspx?Content=Data/RegionalGroupings.

It should be noted that while the UNMDG groups allow for greater differentiation than WHO income and country groupings, they still obscure significant differences between members of specific groups, especially in the case of countries such as Brazil, Nigeria, India, and China. See Additional file 1 for a comprehensive list of mobile phone subscriptions in individual countries.

## Competing interest

We declare that we have no conflicts of interests.

## Authors’ contributions

CF conceived of and drafted the article, drawing on literature in the fields of global mental health and mHealth, and analysing data from the World Health Organization and the World Bank. AA and KR contributed to several subsequent drafts. All authors approved the final manuscript. CF is the guarantor of the article.

## Authors’ information

CF has conducted research in both mental health and eHealth, and is currently carrying out research focused on new medical technologies in a range of fields including mental health, diabetes, and hypertension. AA also has research experience in the mental health field, and has worked with medical technology including medical simulations and eHealth/mental health. KR has worked on health and education policies across public, private, and academic sectors, and has published on e-Learning in healthcare with CF.

## Supplementary Material

Additional file 1 Mental Health DALYs, Mobile Subscriptions by UNMDG Groupings in 2008 and 2012 (millions), Change 2008-2012 (millions; %). Click here for file
