# Supplementary material for: mHealth and global mental health: still waiting for the mH2 wedding?
Source: Global Health. 2014 Mar 26;10:17. doi: 10.1186/1744-8603-10-17 (PMC3986901; doi:10.1186/1744-8603-10-17)
Supplement: Additional file 1 — Mental Health DALYs, Mobile Subscriptions by UNMDG Groupings in 2008 and 2012 (millions), Change 2008-2012 (millions; %). [file 1744-8603-10-17-S1.docx]

**Additional File 1: Mental Health DALYs (2004) and Mobile Phone Subscriptions (2008-2012) by UNMDG Region and Individual Country**

| **UNMDG Region** | **Country** | **Mental Health DALYs (2004)*** | **Mobile Phone Subscriptions in 2008 (millions)**** | **Mobile Phone Subscriptions in 2012 (millions)** | **Change in Mobile Phone Subscriptions between 2008 and 2012 (millions)** | **Change in Mobile Phone Subscriptions between 2008 and 2012 (%)** |
| --- | --- | --- | --- | --- | --- | --- |
| **Developed**  **(n=48)** | Albania | 100 | 1.86 | 3.50 | 1.64 | 46.87 |
|  | Andorra | 2 | 0.06 | 0.07 | 0.00 | 1.23 |
|  | Australia | 612 | 22.12 | 24.34 | 2.22 | 9.11 |
|  | Austria | 265 | 10.82 | 13.59 | 2.77 | 20.40 |
|  | Belarus | 334 | 8.13 | 10.68 | 2.55 | 23.87 |
|  | Belgium | 330 | 11.34 | 12.88 | 1.54 | 11.94 |
|  | Bosnia and Herzegovina | 122 | 3.18 | 3.35 | 0.17 | 5.17 |
|  | Bulgaria | 247 | 10.43 | 10.78 | 0.35 | 3.26 |
|  | Canada | 1 124 | 22.09 | 26.26 | 4.17 | 15.88 |
|  | Croatia | 152 | 4.55 | 4.97 | 0.42 | 8.38 |
|  | Cyprus | 21 | 1.02 | 1.11 | 0.09 | 8.48 |
|  | Czech Republic | 303 | 13.78 | 12.97 | -0.81 | -6.22 |
|  | Denmark | 173 | 6.56 | 6.60 | 0.04 | 0.61 |
|  | Estonia | 47 | 1.62 | 2.07 | 0.45 | 21.54 |
|  | Finland | 194 | 6.38 | 9.32 | 2.94 | 31.55 |
|  | France | 2 085 | 57.97 | 62.28 | 4.31 | 6.92 |
|  | Germany | 2 551 | 105.52 | 107.66 | 2.13 | 1.98 |
|  | Greece | 289 | 13.80 | 13.35 | -0.45 | -3.34 |
|  | Hungary | 359 | 12.22 | 11.58 | -0.64 | -5.57 |
|  | Iceland | 9 | 0.34 | 0.35 | 0.01 | 2.62 |
|  | Ireland | 134 | 5.05 | 4.91 | -0.14 | -2.90 |
|  | Israel | 208 | 8.98 | 9.23 | 0.24 | 2.63 |
|  | Italy | 1 489 | 90.34 | 97.23 | 6.89 | 7.08 |
|  | Japan | 2 687 | 110.39 | 138.36 | 27.97 | 20.21 |
|  | Latvia | 79 | 2.30 | 2.31 | 0.01 | 0.49 |
|  | Lithuania | 119 | 5.02 | 5.00 | -0.03 | -0.51 |
|  | Luxembourg | 15 | 0.71 | 0.76 | 0.05 | 7.13 |
|  | Macedonia | 63 | 1.97 | 2.24 | 0.27 | 11.99 |
|  | Malta | 11 | 0.39 | 0.54 | 0.15 | 28.51 |
|  | Moldova | 140 | 2.42 | 4.08 | 1.66 | 40.60 |
|  | Monaco | 1 | 0.02 | 0.03 | 0.01 | 33.72 |
|  | Netherlands | 490 | 20.63 | 19.64 | -0.98 | -5.01 |
|  | New Zealand | 110 | 4.62 | 4.92 | 0.30 | 6.14 |
|  | Norway | 162 | 5.21 | 5.73 | 0.52 | 9.08 |
|  | Poland | 1 235 | 42.93 | 50.84 | 7.91 | 15.57 |
|  | Portugal | 312 | 14.05 | 12.31 | -1.74 | -14.11 |
|  | Romania | 686 | 24.47 | 22.70 | -1.77 | -7.80 |
|  | Russian Federation | 5 810 | 199.52 | 261.89 | 62.36 | 23.81 |
|  | San Marino | 1 | 0.02 | 0.04 | 0.01 | 32.22 |
|  | Serbia | 343 | 9.62 | 9.14 | -0.48 | -5.26 |
|  | Slovak Republic | 198 | 5.52 | 6.09 | 0.57 | 9.43 |
|  | Slovenia | 66 | 2.05 | 2.25 | 0.19 | 8.49 |
|  | Spain | 1 181 | 49.62 | 50.66 | 1.04 | 2.05 |
|  | Sweden | 305 | 10.01 | 11.64 | 1.63 | 13.99 |
|  | Switzerland | 241 | 8.90 | 10.64 | 1.74 | 16.38 |
|  | Ukraine | 1 543 | 55.68 | 59.34 | 3.66 | 6.17 |
|  | United Kingdom | 2 058 | 76.74 | 82.11 | 5.37 | 6.54 |
|  | United States | 11 709 | 261.30 | 310.00 | 48.70 | 15.71 |
|  | **Average (developed region)** | **848.23** | **27.76** | **31.72** | **3.96** | **10.15** |
|  | **Total (developed region)** | **40 715** | **1 332.29** | **1 522.33** | **190.04** | **487.06** |
| **All regions other than Developed region**  **(n=144)** | **Average***** | **17 747.87** | **42.79** | **65.13** | **25.07** | **46.29** |
|  | **Total** | **161 184.85** | **2 652.62** | **4 735.94** | **1 451.68** | **6 200.59** |
| **Northern Africa**  **(n=6)** | Algeria | 800 | 27.03 | 37.69 | 10.66 | 28.28 |
|  | Egypt | 2 220 | 41.29 | 96.80 | 55.51 | 57.35 |
|  | Libya | 163 | 7.38 | 9.59 | 2.21 | 23.03 |
|  | Morocco | 875 | 22.82 | 39.02 | 16.20 | 41.52 |
|  | Tunisia | 276 | 8.60 | 12.84 | 4.24 | 33.01 |
|  | Western Sahara | N/A | N/A | N/A | N/A | N/A |
|  | **Average (Northern Africa)** | **866.8** | **21.42** | **39.19** | **17.76** | **36.64** |
|  | **Total (Northern Africa)** | **4 334** | **107.12** | **195.94** | **88.82** | **183.20** |
| **Sub-Saharan Africa**  **(n=48)** | Angola | 437 | 6.77 | 9.80 | 3.03 | 30.89 |
|  | Benin | 210 | 3.63 | 8.41 | 4.78 | 56.88 |
|  | Botswana | 46 | 1.49 | 3.08 | 1.60 | 51.79 |
|  | Burkina Faso | 364 | 3.02 | 9.98 | 6.95 | 69.69 |
|  | Burundi | 199 | 0.48 | 2.25 | 1.77 | 78.61 |
|  | Cameroon | 460 | 6.16 | 13.11 | 6.95 | 53.00 |
|  | Cape Verde Islands | 12 | 0.28 | 0.43 | 0.15 | 34.71 |
|  | Central African Republic | 102 | 0.25 | 1.07 | 0.82 | 76.64 |
|  | Chad | 261 | 1.60 | 4.20 | 2.60 | 61.89 |
|  | Coromos | 19 | 0.09 | 0.25 | 0.16 | 63.30 |
|  | Congo (Republic of) | 85 | 1.81 | 4.82 | 3.02 | 62.53 |
|  | Cote d'Ivoire | 1 477 | 10.45 | 19.83 | 9.38 | 47.30 |
|  | Democratic Republic of the Congo | 1 421 | 9.94 | 19.49 | 9.55 | 49.00 |
|  | Djibouti | 21 | 0.11 | 0.21 | 0.10 | 46.01 |
|  | Equatorial Guinea | 12 | 0.18 | 0.50 | 0.32 | 64.08 |
|  | Eritrea | 106 | 0.11 | 0.31 | 0.20 | 64.42 |
|  | Ethiopia | 1 856 | 1.95 | 20.52 | 18.57 | 90.48 |
|  | Gabon | 33 | 1.30 | 2.93 | 1.63 | 55.63 |
|  | Gambia, The | 40 | 1.17 | 1.53 | 0.36 | 23.59 |
|  | Ghana | 663 | 11.57 | 25.62 | 14.05 | 54.84 |
|  | Guinea | 223 | 2.75 | 4.78 | 2.03 | 42.48 |
|  | Guinea-Bissau | 40 | 0.50 | 1.10 | 0.60 | 54.41 |
|  | Kenya | 854 | 16.60 | 30.73 | 14.13 | 45.97 |
|  | Lesotho | 47 | 0.59 | 1.31 | 0.72 | 54.78 |
|  | Liberia | 88 | 0.85 | 2.39 | 1.54 | 64.30 |
|  | Madagascar | 547 | 4.84 | 8.56 | 3.73 | 43.54 |
|  | Malawi | 304 | 1.51 | 4.42 | 2.91 | 65.89 |
|  | Mali | 293 | 3.44 | 14.61 | 11.17 | 76.47 |
|  | Mauritania | 72 | 2.09 | 4.02 | 1.93 | 48.01 |
|  | Mauritius | 32 | 1.03 | 1.49 | 0.45 | 30.42 |
|  | Mozambique | 484 | 4.41 | 8.11 | 3.70 | 45.67 |
|  | Namibia | 47 | 1.05 | 2.44 | 1.38 | 56.80 |
|  | Niger | 327 | 1.90 | 5.40 | 3.50 | 64.83 |
|  | Nigeria | 4 124 | 62.99 | 112.78 | 49.79 | 44.15 |
|  | Rwanda | 233 | 1.32 | 5.69 | 4.37 | 76.76 |
|  | Sao Tome and Principe | 4 | 0.05 | 0.12 | 0.07 | 58.56 |
|  | Senegal | 285 | 5.39 | 11.47 | 6.08 | 53.02 |
|  | Seychelles | 2 | 0.09 | 0.14 | 0.04 | 32.40 |
|  | Sierra Leone | 146 | 1.01 | 2.21 | 1.20 | 54.35 |
|  | Somalia | 224 | 0.63 | 0.66 | 0.03 | 4.71 |
|  | South Africa | 1 320 | 45.00 | 68.39 | 23.39 | 34.20 |
|  | Sudan | 909 | 11.99 | 27.66 | 15.67 | 56.64 |
|  | Swaziland | 29 | 0.53 | 0.81 | 0.27 | 33.96 |
|  | Togo | 155 | 1.55 | 3.52 | 1.97 | 55.95 |
|  | Uganda | 693 | 8.55 | 16.36 | 7.80 | 47.70 |
|  | Tanzania | 921 | 13.01 | 27.22 | 14.21 | 52.21 |
|  | Zambia | 282 | 3.54 | 10.52 | 6.99 | 66.37 |
|  | Zimbabwe | 340 | 1.65 | 12.61 | 10.96 | 86.88 |
|  | **Average (Sub-Saharan Africa)** | **434.3542** | **5.44** | **11.20** | **5.76** | **53.89** |
|  | **Total (Sub-Saharan Africa** | **20 849** | **261.23** | **537.83** | **276.61** | **2 586.72** |
| **Latin America and the Caribbean**  **(n=33)** | Antigua and Barbuda | 3 | 0.06 | 0.18 | 0.12 | 64.29 |
|  | Argentina | 1 450 | 46.51 | 58.60 | 12.09 | 20.63 |
|  | Bahamas, The | 12 | 0.36 | 0.25 | -0.10 | -40.96 |
|  | Barbados | 11 | 0.29 | 0.35 | 0.06 | 16.81 |
|  | Belize | 10 | 0.16 | 0.16 | 0.00 | 2.51 |
|  | Bolivia | 356 | 5.04 | 9.49 | 4.46 | 46.93 |
|  | Brazil | 7 711 | 150.64 | 248.32 | 97.68 | 39.34 |
|  | Chile | 644 | 14.80 | 24.13 | 9.33 | 38.68 |
|  | Colombia | 1 898 | 41.36 | 49.07 | 7.70 | 15.70 |
|  | Costa Rica | 151 | 1.89 | 6.15 | 4.26 | 69.33 |
|  | Cuba | 372 | 0.33 | 1.68 | 1.35 | 80.27 |
|  | Dominica | 3 | 0.09 | 0.11 | 0.02 | 16.74 |
|  | Dominican Republic | 335 | 7.21 | 9.04 | 1.83 | 20.22 |
|  | Ecuador | 521 | 11.68 | 16.46 | 4.77 | 29.00 |
|  | El Salvador | 253 | 6.95 | 8.65 | 1.70 | 19.64 |
|  | Grenada | 4 | 0.06 | 0.13 | 0.07 | 53.11 |
|  | Guatemala | 467 | 14.95 | 20.79 | 5.84 | 28.09 |
|  | Guyana | 27 | 0.45 | 0.55 | 0.10 | 18.15 |
|  | Haiti | 352 | 3.20 | 6.09 | 2.89 | 47.50 |
|  | Honduras | 236 | 6.21 | 7.37 | 1.16 | 15.73 |
|  | Jamaica | 93 | 2.72 | 2.67 | -0.06 | -2.16 |
|  | Mexico | 3 155 | 75.30 | 100.79 | 25.48 | 25.28 |
|  | Nicaragua | 199 | 3.11 | 5.35 | 2.24 | 41.86 |
|  | Panama | 110 | 3.92 | 6.77 | 2.85 | 42.16 |
|  | Paraguay | 200 | 5.79 | 6.79 | 1.00 | 14.76 |
|  | Peru | 1118 | 20.95 | 29.39 | 8.44 | 28.71 |
|  | St. Kitts and Nevis | 2 | 0.07 | n/a | n/a | n/a |
|  | St. Lucia | 6 | 0.18 | n/a | n/a | n/a |
|  | St. Vincent and the Grenadines | 5 | 0.13 | n/a | n/a | n/a |
|  | Trinidad and Tobago | 48 | 1.81 | 1.88 | 0.08 | 4.12 |
|  | Suriname | 16 | 0.66 | 0.98 | 0.32 | 32.74 |
|  | Uruguay | 124 | 3.51 | 5.00 | 1.49 | 29.78 |
|  | Venezuela | 1034 | 27.41 | 30.52 | 3.10 | 10.17 |
|  | **Average (Latin America and the Caribbean)** | **634.1212** | **13.87** | **21.92** | **6.68** | **27.64** |
|  | **Total (Latin America and the Caribbean)** | **20 926** | **457.80** | **657.70** | **200.27** | **829.12** |
| **Caucasus and Central Asia**  **(n=8)** | Armenia | 89 | 1.44 | 3.32 | 1.88 | 56.60 |
|  | Azerbaijan | 262 | 6.55 | 10.13 | 3.58 | 35.33 |
|  | Georgia | 158 | 2.76 | 4.70 | 1.94 | 41.36 |
|  | Kazakhstan | 549 | 14.91 | 28.73 | 13.82 | 48.10 |
|  | Kyrgyz Republic | 166 | 3.39 | 6.80 | 3.40 | 50.07 |
|  | Tajikistan | 209 | 3.67 | 6.53 | 2.85 | 43.73 |
|  | Turkmenistan | 147 | 1.14 | 3.95 | 2.82 | 71.28 |
|  | Uzbekistan | 823 | 12.38 | 20.27 | 7.90 | 38.96 |
|  | **Average (Caucasus and Central Asia)** | **300.375** | **5.78** | **10.55** | **4.77** | **48.18** |
|  | **Total (Caucasus and Central Asia)** | **2403** | **46.23** | **84.43** | **38.20** | **385.44** |
| **Eastern Asia**  **(n=4)** | China | 36 878 | 641.25 | 1100.00 | 458.76 | 41.71 |
|  | Korea, Dem.Rep. | 628 | n/a | 1.70 | n/a | n/a |
|  | Korea, Rep. | 1614 | 1.76 | 3.38 | 1.61 | 47.76 |
|  | Mongolia | 76 | 45.61 | 53.62 | 8.02 | 14.95 |
|  | **Average (Eastern Asia)** | **9799** | **229.54** | **289.67** | **156.13** | **34.81** |
|  | **Total (Eastern Asia)** | **39 196** | **688.62** | **1 158.70** | **468.38** | **104.42** |
| **Southern Asia**  **(n=9)** | Afghanistan | 898 | 7.90 | 18.00 | 2.28 | 12.66 |
|  | Bangladesh | 4 611 | 44.64 | 97.18 | 2.18 | 2.24 |
|  | Bhutan | 20 | 0.25 | 0.56 | 2.21 | 394.59 |
|  | India | 35 981 | 346.89 | 864.72 | 2.49 | 0.29 |
|  | Iran | 2363 | 43.00 | 58.16 | 1.35 | 2.33 |
|  | Maldives | 11 | 0.44 | 0.56 | 1.29 | 229.55 |
|  | Nepal | 866 | 4.20 | 16.38 | 3.90 | 23.81 |
|  | Pakistan | 4 895 | 88.02 | 120.15 | 1.37 | 1.14 |
|  | Sri Lanka | 555 | 11.08 | 20.32 | 1.83 | 9.02 |
|  | **Average (Southern Asia)** | **5 577.778** | **60.71** | **132.89** | **2.10** | **75.07** |
|  | **Total (Southern Asia)** | **50 200** | **546.42** | **1 196.03** | **18.90** | **675.62** |
| **South-Eastern Asia**  **(n=11)** | Brunei Darussalam | 9 | 0.40 | 0.47 | 0.07 | 15.09 |
|  | Cambodia | 451 | 4.24 | 19.11 | 14.87 | 77.82 |
|  | Indonesia | 6 121 | 140.58 | 281.96 | 141.39 | 50.14 |
|  | Lao | 189 | 2.02 | 6.49 | 4.47 | 68.85 |
|  | Malaysia | 708 | 27.71 | 41.32 | 13.61 | 32.94 |
|  | Myanmar | 1 368 | 0.37 | 5.44 | 5.07 | 93.25 |
|  | Philippines | 2 562 | 68.12 | 103.00 | 34.88 | 33.87 |
|  | Singapore | 101 | 6.41 | 8.06 | 1.65 | 20.44 |
|  | Thailand | 2 087 | 61.84 | 84.08 | 22.24 | 26.45 |
|  | Timor-Leste | 29 | 0.13 | 0.62 | 0.50 | 79.87 |
|  | Vietnam | 2 373 | 74.87 | 134.07 | 59.19 | 44.15 |
|  | **Average (South-Eastern Asia)** | **1 454.364** | **35.15** | **62.24** | **27.09** | **49.35** |
|  | **Total (South-Eastern Asia)** | **17 452.36** | **386.68** | **684.62** | **297.94** | **542.87** |
| **Western Asia**  **(n=12)** | Bahrain | 20 | 1.44 | 2.12 | 0.68 | 32.16 |
|  | Iraq | 866 | 17.53 | 26.76 | 9.23 | 34.49 |
|  | Jordan | 150 | 5.31 | 8.98 | 3.67 | 40.86 |
|  | Kuwait | 66 | 1.45 | 5.53 | 4.08 | 73.76 |
|  | Lebanon | 109 | 1.43 | 4.00 | 2.57 | 64.33 |
|  | Oman | 64 | 3.22 | 5.28 | 2.06 | 39.00 |
|  | Qatar | 20 | 1.43 | 2.60 | 1.17 | 45.02 |
|  | Saudi Arabia | 602 | 36.00 | 53.01 | 17.01 | 32.09 |
|  | Syrian Arab Republic | 507 | 7.06 | 12.93 | 5.87 | 45.42 |
|  | Turkey | 2 049 | 65.82 | 67.68 | 1.86 | 2.74 |
|  | United Arab Emirates | 104 | 9.36 | 13.78 | 4.42 | 32.07 |
|  | Yemen | 615 | 6.45 | 13.90 | 7.46 | 53.63 |
|  | **Average (Western Asia)** | **431** | **13.04** | **18.05** | **5.01** | **41.30** |
|  | **Total (Western Asia)** | **5 603** | **156.49** | **216.56** | **60.07** | **495.57** |
| **Oceania**  **(n=13)** | Fiji | 22.00 | 0.60 | 0.86 | 0.26 | 30.14 |
|  | Kiribati | 2.00 | 0.00 | 0.02 | 0.02 | 93.75 |
|  | Marshall Islands | 1.00 | n/a | n/a | n/a | n/a |
|  | Micronesia, Fed.Sts. | 3.00 | 0.03 | 0.03 | 0.00 | 0.36 |
|  | Nauru | 0.00 | n/a | n/a | n/a | n/a |
|  | Niue | 0.00 | n/a | n/a | n/a | n/a |
|  | Palau | 0.00 | 0.01 | 0.02 | 0.01 | 31.97 |
|  | Papua New Guinea | 155.00 | 0.87 | 2.71 | 1.84 | 67.74 |
|  | Samoa | 4.00 | n/a | n/a | n/a | n/a |
|  | Solomon Islands | 12.00 | 0.03 | 0.30 | 0.27 | 90.07 |
|  | Tonga | 2.00 | 0.05 | 0.06 | 0.01 | 9.87 |
|  | Tuvalu | 0.00 | n/a | 0.00 | n/a | n/a |
|  | Vanuatu | 5.00 | 0.04 | 0.14 | 0.10 | 73.72 |
|  | **Average (Oceania)** | **15.85** | **0.20** | **0.46** | **0.31** | **49.70** |
|  | **Total (Oceania)** | **206.00** | **1.63** | **4.13** | **2.49** | **397.63** |

*2004 figures from World Health Organization.^[4]^

**2012 figures from World Bank.^[23]^

*** Averages are of statistics across non-developed region UNMDG groups; not weighted by population size or number of constituent countries in each group.
